# Radiocarbon evidence for enhanced respired carbon storage in the Atlantic at the Last Glacial Maximum

**DOI:** 10.1038/ncomms11998

**Published:** 2016-06-27

**Authors:** E. Freeman, L. C. Skinner, C. Waelbroeck, D. Hodell

**Affiliations:** 1 Godwin Laboratory for Palaeoclimate Research, Department of Earth Sciences, University of Cambridge, Cambridge CB2 3EQ, UK; 2 LSCE/IPSL, Laboratoire CNRS-CEA-UVSQ, Domaine du CNRS, bât 12 91198 Gif-sur-Yvette, France

## Abstract

The influence of ocean circulation changes on atmospheric CO_2_ hinges primarily on the ability to alter the ocean interior's respired nutrient inventory. Here we investigate the Atlantic overturning circulation at the Last Glacial Maximum and its impact on respired carbon storage using radiocarbon and stable carbon isotope data from the Brazil and Iberian Margins. The data demonstrate the existence of a shallow well-ventilated northern-sourced cell overlying a poorly ventilated, predominantly southern-sourced cell at the Last Glacial Maximum. We also find that organic carbon remineralization rates in the deep Atlantic remained broadly similar to modern, but that ventilation ages in the southern-sourced overturning cell were significantly increased. Respired carbon storage in the deep Atlantic was therefore enhanced during the last glacial period, primarily due to an increase in the residence time of carbon in the deep ocean, rather than an increase in biological carbon export.

## Abstract (web summary)

The Atlantic Ocean overturning circulation and its impact on respired carbon storage during glacial periods remains a mystery. Here, the authors combine radiocarbon and stable carbon isotope data from the Brazil and Iberian Margins to investigate ocean circulation and carbon storage at the Last Glacial Maximum.

## Graphical abstract

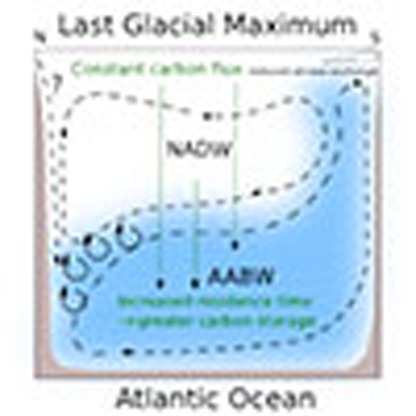

Numerous proxies suggest that ocean circulation at the Last Glacial Maximum (LGM) was different from modern, especially in the Atlantic Ocean^1^,^2^,^3^,^4^. Despite this, a consensus has yet to emerge on the LGM ocean circulation^5^, primarily because of limitations associated with the various proxies, as well as a paucity of data. In agreement with stable oxygen isotope data^6^, δ^13^C (stable carbon isotopic ratio) and Cd/Ca data suggest that North Atlantic Deep Water (NADW) shoaled by up to 1,000 m during the LGM relative to today^3^,^4^,^7^. However, the combination of conservative and non-conservative impacts on nutrient proxies has permitted alternative interpretations, such as greater nutrient accumulation along the flow path^8^ (because of greater export production or longer transit times), or variable contributions from northern- and southern-sourced waters between 2 and 4 km water depth^9^ (without a complete elimination of NADW in the deep Atlantic). Although ^231^Pa/^230^Th has been used to reconstruct past rates of overturning circulation^10^,^11^, its interpretation presents difficulties, as it represents an integrated flow speed across the water column and may be affected by scavenging^12^.

Additional proxy data are therefore needed to reconstruct the pattern and rate of ocean circulation at the LGM, and to assess its impact on the marine carbon cycle. Radiocarbon-based ventilation ages could prove particularly useful in this regard as they provide an indication of the mean timescale for carbon exchange between the ocean and the atmosphere, including the effects of air–sea gas (and isotope) exchange in the regions of origin of a given parcel of water, and the mean time elapsed since that water was in the mixed layer. Furthermore, by combining ventilation ages with indicators of respired carbon accumulation, such as stable carbon isotopes, changes in the efficiency of the biological carbon pump can be estimated. This is not possible using reconstructions of flow rates or respired/total nutrient levels alone, as the efficiency of the biological carbon pump is controlled by both the export productivity (that is, the flux of carbon into the deep ocean) and the ‘leakage' of this carbon out of the deep ocean (via all the processes that contribute to mass transfer from the deep ocean interior to the surface ocean mixed layer where air–sea exchange occurs).

Here we investigate the Atlantic Ocean overturning circulation at the LGM using both radiocarbon and stable carbon isotopes. We find a shallow well-ventilated northern-sourced cell overlying a poorly ventilated, predominantly southern-sourced cell. Furthermore, we find that the storage of respired carbon in the deep Atlantic Ocean was enhanced because of a long residence time, with a constant flux of respired carbon to the deep Atlantic.

## Results

### Radiocarbon and stable carbon isotopes

Here we present new radiocarbon-based ventilation ages along with stable carbon isotopes from 1,000 to 3,500 m on the Brazil Margin and from 1,100 to 4,700 m on the Iberian Margin (Supplementary Table 1). Ventilation ages are based on benthic-planktonic (B-P) radiocarbon age offsets, to which the estimated surface reservoir age is added to provide an estimate of the deep ocean versus atmospheric radiocarbon age offset (B-Atm). Given the proximity of cores in each of our study regions, surface reservoir ages are expected to be very similar for all cores in each transect. Therefore, despite some uncertainty in the surface reservoir ages, vertical gradients in ventilation ages within each transect will remain accurate.

Radiocarbon-based ventilation ages provide an indication of the extent of isotopic disequilibrium between the marine- and atmospheric carbon reservoirs. More radiocarbon-depleted waters (indicated by higher radiocarbon ventilation ages) will reflect less efficient radiocarbon (and therefore CO_2_) exchange between the ocean interior and the atmosphere on average, and therefore a longer carbon sequestration time in the ocean interior. For continuous export of biologically fixed carbon to the ocean interior, a greater radiocarbon ventilation age and a longer carbon sequestration time (that is, relative to the atmosphere) should in turn reflect enhanced accumulation of respired carbon in the ocean interior and a more efficient (since less ‘leaky') soft-tissue carbon pump^13^.

### Ventilation of the Brazil Margin

**Figure 1:**
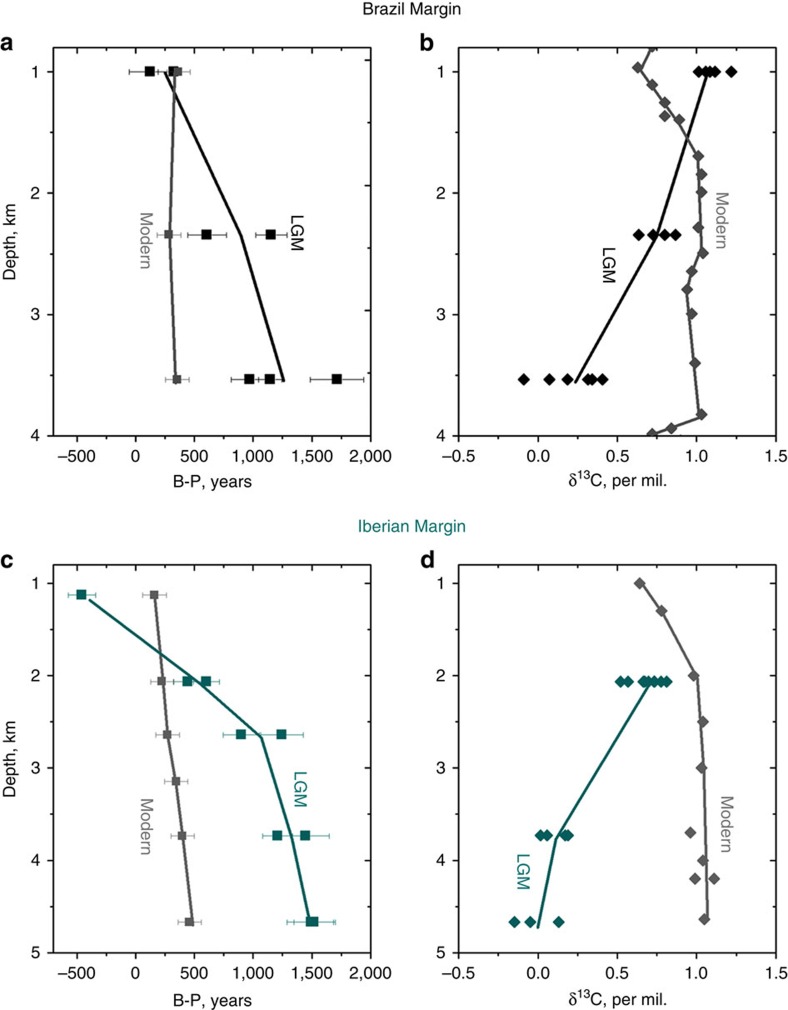
LGM depth profiles for the Brazil Margin and the Iberian Margin. The benthic-planktonic radiocarbon age offset (B-P; **a**, **c**) and the stable carbon isotopic ratio, δ^13^C (**b**, **d**). Modern B-P values (calculated by subtracting the modern surface reservoir age from the bottom water ventilation age) are show in grey (GLODAP^38^). Modern δ^13^C of DIC is also shown in grey (GEOSECS^38^). Data are given in Supplementary Table 3.

On the Brazil Margin, the radiocarbon ventilation age-depth profile for the LGM is significantly different from that of the modern ocean (Fig. 1). Although the shallowest core (1,000 m), has a similar ventilation age to that of the modern ocean, the deeper cores have considerably higher ventilation ages, with the deepest core being offset from the surface ocean by up to 1,700 years, compared with just 400 years today.

Stable carbon isotopes also had a distinctly different profile at the LGM compared with the modern. δ^13^C shows a significant decrease with depth, ranging from ∼1.1‰ at 1,000 m to ∼0.2‰ in the deep ocean. The steepest gradient (∼−0.5‰/1,000 m) is seen between 2,300 and 3,550 m, whereas in the modern ocean the gradient is <0.02‰/1,000 m between these depths.

### Ventilation of the Iberian Margin

The Iberian Margin also shows a very different ventilation age depth-profile at the LGM compared with the modern ocean (Fig. 1). Although the intermediate ocean (<2,100 m) was well ventilated, the deep ocean was poorly ventilated, with B-P age offsets more than 1,000 years greater than in the modern ocean. The shallowest core (1,127 m) has a negative B-P age offset suggesting that the surface was more poorly ventilated than the intermediate ocean (likely influenced by Mediterranean Outflow Water^14^). Beneath this, the B-P age offset increases with depth in the water column. A relatively steep gradient (∼950 years/1,000 m) is seen for the upper 2,600 m, with a smaller increase in B-P age offset with depth below (∼200 years/1,000 m). A similar profile is also seen in δ^13^C with a steep gradient (∼−0.4‰/1,000 m) between 2 and 3.7 km but with relatively little change (<−0.1‰/1,000 m) with depth below ∼3,700 m. The range of δ^13^C (0–0.8‰) at the LGM is significantly greater than the range in the modern North Atlantic (1.0–1.1‰) between 2 and 4.7 km. δ^13^C at 4.7 km are more than 0.9‰ lower at the LGM than in the modern ocean. However, extremely low δ^13^C, previously observed in both the deep South Atlantic (<−0.8‰)^15^,^16^ and in the abyssal western North Atlantic (−0.3 to −0.5‰)^17^,^18^, are not observed in any of the Iberian Margin cores.

## Discussion

**Figure 2:**
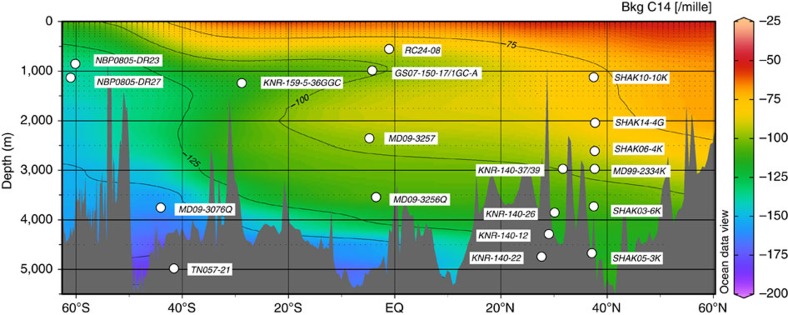
Map of core locations. Map showing core/dredge locations with modern background Δ^14^C (GLODAP^38^), using Ocean Data View^71^.

**Figure 3:**
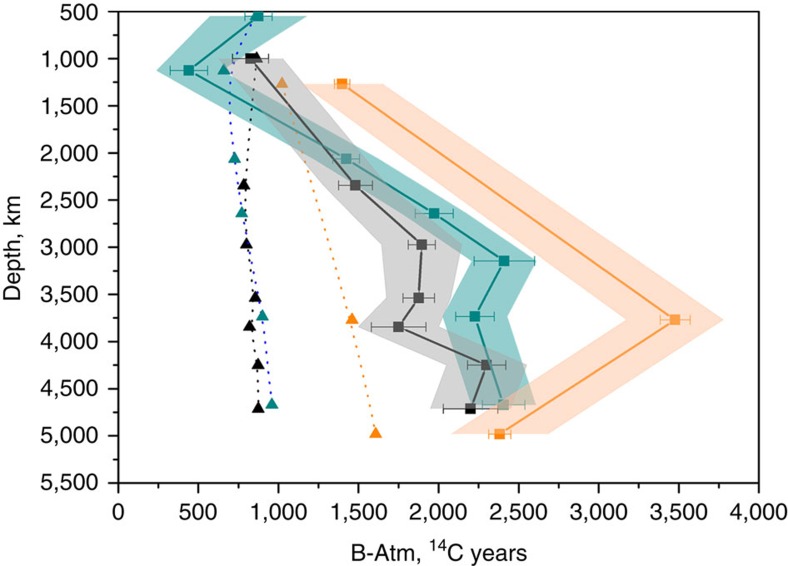
Atlantic radiocarbon-based ventilation age depth profiles. Triangles: modern ventilation ages (dotted lines). Squares: LGM ventilation ages (solid lines)^18^,^19^,^20^,^21^,^22^,^23^,^24^,^34^. Error bars indicate the analytical error on the radiocarbon measurements. The shaded area represents the uncertainty of the surface reservoir age. Blue, East Atlantic; black, West Atlantic; orange, South Atlantic.

**Figure 4:**
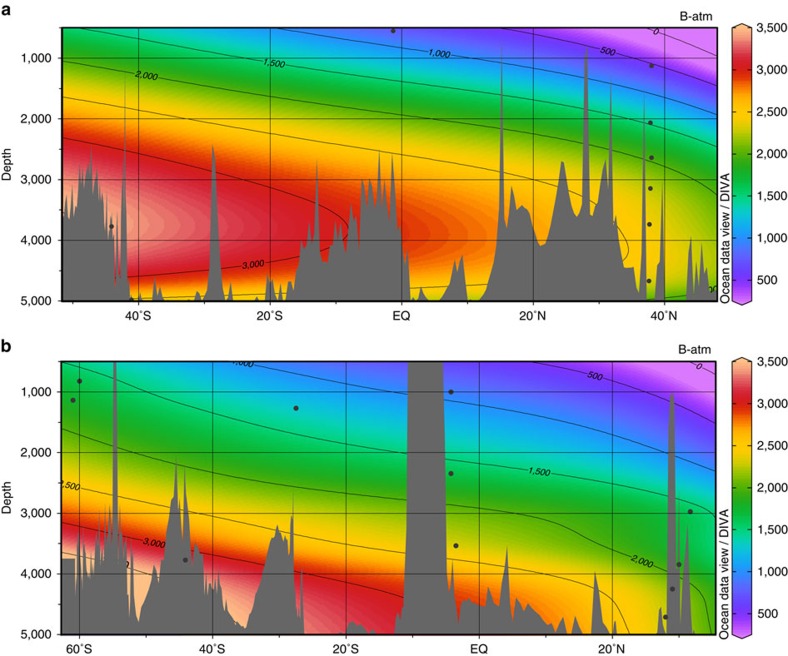
Atlantic radiocarbon based ventilation ages at the LGM. (**a**) Eastern Atlantic. (**b**) Western Atlantic^18^,^19^,^20^,^21^,^22^,^23^,^24^,^25^,^34^. MD07-3076Q (44°S, 14°W, 3770, m) is included in both the eastern and the western profiles as it is located on the Mid-Atlantic Ridge. The dots indicate the position of the cores where ventilation ages have been determined. Gridded in ODV^71^ using DIVA gridding with a signal-to-noise ratio of 72. Data are given in Supplementary Table 4.

We combined our new ventilation ages with available published data in order to tentatively reconstruct the distribution of radiocarbon in the Atlantic Ocean during the LGM^18^,^19^,^20^,^21^,^22^,^23^,^24^,^25^ (Fig. 2 and Supplementary Table 2). These radiocarbon-based ventilation ages show that, regardless of the uncertainties in LGM surface reservoir ages, the entire deep Atlantic Ocean (>2 km) was less ventilated during the LGM than the modern ocean (Fig. 3). Today, the deep North Atlantic is well ventilated by newly formed NADW, whereas the deep South Atlantic is primarily influenced by southern-sourced waters (Lower Circumpolar Deep Water, LCDW, and Antarctic Bottom Water, AABW), which have greater ventilation ages because of reduced air–sea gas exchange in the AABW formation regions^26^ and because of mixing and entrainment of ‘pre-aged' circumpolar deep water into AABW^27^. During the LGM this North–South ventilation age gradient in the deep Atlantic was significantly reduced or even eradicated (in the abyss), suggesting a distinctly different circulation must have prevailed (Figs 3 and 4). Based on the apparent northward expansion of poorly ventilated deep waters from the glacial South Atlantic to the deep equatorial and North Atlantic, the LGM circulation appears to have been characterized primarily by a greatly reduced influence of NADW in the deepest Atlantic (>2,000 m). It is notable that abyssal waters in the western North Atlantic and on the northern Brazil Margin had a similar ventilation age to that of the abyssal South Atlantic (Fig. 3). The lack of an increase in ventilation age along the flow path between the South Atlantic and the equatorial and North Atlantic suggests that neither the deep eastern Atlantic nor the deep western Atlantic contained pure southern-sourced waters. A persistent, albeit greatly reduced, northern source of radiocarbon was therefore provided to depths of at least 4.7 km, either via mixing with an overlying well-ventilated water mass or via direct episodic input of dense well-ventilated (for example, overflow) waters.

**Figure 5:**
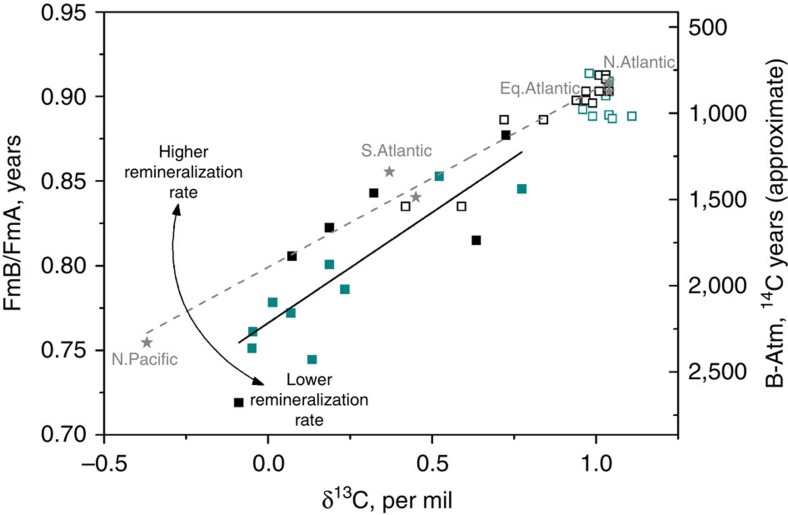
Crossplot of ventilation age and stable carbon isotopic ratio. Ventilation ages are plotted as FmB/FmA, where FmB/FmA=exp(B-Atm/-8033), and Fm=fraction modern, because B-Atm ages do not mix linearly due to the exponential decay of radiocarbon. Approximate B-Atm values are shown as a guideline only. Modern whole ocean data (∼2.5 km water depth; grey stars) is show along with modern data for the entire water column >2 km (open squares) on the Iberian Margin (blue) and Brazil Margin (black). LGM data are shown (filled squares) for the Iberian Margin (blue) and the Brazil Margin (black; Modern data are from GEOSECS and GLODAP^38^). The modern gradient is 0.11±0.01 and the LGM gradient is 0.13±0.02, equivalent to 1.1±0.1‰/kyr and 0.9±0.2‰/kyr in the modern and in the LGM, respectively. For a given rate of remineralization, an increase in residence time (that is, lower FmB/FmA) results in a greater amount of carbon storage.

In the modern ocean, radiocarbon-based ventilation ages are well correlated with DIC and anti-correlated with δ^13^C owing to the remineralization of organic carbon as waters flow from the North Atlantic to the North Pacific, via the Southern Ocean (Fig. 5). DIC thus increases in the modern ocean by ∼115 μmol kg^−1^ (ref. 13), whereas the δ^13^C of DIC decreases by ∼0.8‰ (Fig. 5), for every 10% decrease in the deep ocean's radiocarbon content relative to the atmosphere (that is, for every ∼850 ^14^Cyr ventilation age increase). A similar gradient in δ^13^C versus ventilation age is seen in the North and equatorial Atlantic at the LGM, suggesting that the remineralization rate of organic carbon in the deep Atlantic did not change significantly. On this basis, the LGM rate of DIC increase per year of radiocarbon decay (because of the soft-tissue pump alone) would also be expected to have remained roughly constant, as hypothesized in ref. 13. All else being equal, the ventilation age increase that we observe across the deep Atlantic would therefore imply a significant increase in the respired carbon inventory of the deep ocean at the LGM. This increase was proposed in ref. 8 but later questioned in ref. 9 because of an updated LGM state estimate. Here we not only propose an increase in the remineralized carbon inventory of the deep ocean (based on stable carbon isotope evidence, similar to that advanced previously), but also attribute it to an increase in residence time rather than an increase in biological carbon export rates.

Our results demonstrate a distinct difference in the distribution of radiocarbon in the Atlantic at the LGM relative to today. The deep ocean was more poorly ventilated at all latitudes, whereas the intermediate ocean remained as well ventilated as in the modern ocean. This strongly suggests a shoaling of northern-sourced waters, without a major reduction in their overturning strength, and an expansion of southern-sourced waters at depth. This is in agreement with other proxy reconstructions, which our radiocarbon data help to link more directly to carbon cycle impacts. The concentration of dissolved cadmium inferred from Cd/Ca measured on benthic foraminifera show a clear decrease in the upper ∼2.2 km of the water column at the LGM relative to today, with the opposite sense of change in the deep ocean^4^ (Supplementary Fig. 1a). This would suggest that high-nutrient waters expanded at depth while low-nutrient northern-sourced waters dominated in the intermediate ocean, consistent with δ^13^C data that also show the opposite sense of change above and below ∼2.2 km (ref. 4; Supplementary Fig. 1b). In principle, this change in nutrient distributions could have been driven by enhanced export productivity and/or longer ocean interior residence times. As indicated above, our combined radiocarbon and stable isotope data indicate that the latter mechanism was dominant. Our interpretation that this change in circulation was primarily associated with an expansion of southern-sourced waters in the deepest Atlantic is further supported by ɛNd data, which indicate an increase in southern-sourced waters at depth with an increase in northern-sourced waters above^28^ (Supplementary Fig. 1c). ^231^Pa/^230^Th ratios also display a distinct change of the opposite sign above and below ∼2.2 km water depth^10^ (Supplementary Fig. 1d). These ^231^Pa/^230^Th ratios suggest that the North Atlantic overturning circulation cell was just as strong during the LGM as it is today, but that it penetrated to shallower depths. Although this contrasts with many numerical modelling studies that tend to find a slower overturning when NADW shoals^29^,^30^, our ventilation ages also suggest that the upper North Atlantic overturning rate was not reduced so much that it significantly decreased the supply radiocarbon to the intermediate ocean. The radiocarbon data are therefore consistent with the proposal that a significant shoaling of NADW may not necessarily imply a major reduction in the NADW formation/transport rate at the LGM.

However, while our results support the existence at the LGM of a shallow NADW cell overlying an expanded, radiocarbon-depleted AABW below, they also suggest that the deep southern-sourced cell was not completely isolated. In both the eastern and the western North Atlantic, a northern source of relatively radiocarbon-enriched and high δ^13^C waters was mixed into the deep cell. This could imply a more complex Atlantic circulation than one characterized by two completely separate overturning cells at the LGM^31^, where the northern overturning cell is either strong and deep or weak and shallow.

As in the landmark study of ref. 3, our stable isotope data demonstrate the existence of enhanced gradients in δ^13^C between ∼2,000 and 3,500 m in the equatorial and North Atlantic. This pattern of δ^13^C in the Atlantic has been interpreted primarily to demonstrate a reduction in the proportion of northern-sourced waters with depth, and an increase in the total nutrient content of the deep Atlantic. Our new radiocarbon data demonstrate that this did not primarily reflect an increase in ‘preformed' nutrients (that is, nutrients that were advected to the ocean interior, and that would be associated with carbon that was well-equilibrated with the atmosphere^32^), but rather an increase in respired nutrients (and therefore respired carbon). Alternatively, and equivalently for ocean versus atmosphere carbon inventory changes, the radiocarbon data might also indicate an increase in the ‘disequilibrium' carbon inventory of the deep Atlantic^33^. However, in this case, a very large decrease in air–sea CO_2_ exchange efficiency would need to be invoked in parallel with an increase in preformed nutrients, in order to account for the paired stable carbon isotope (and nutrient) reconstructions. The observed expansion of southern-sourced deep waters at the LGM was therefore very likely associated with an increase in the efficiency of the soft-tissue carbon pump, primarily due to an increase in the residence time of carbon in the deep Atlantic, rather than an increase in biological export productivity (that is, at low/mid-latitudes). Although global data coverage and better constraints on surface reservoir ages are required to assess the precise magnitude of the atmospheric CO_2_ drawdown that is implied by the observed ocean circulation changes, our findings support a direct contribution from the Atlantic overturning circulation via its impact on the deep ocean's respired carbon inventory.

## Methods

### Radiocarbon and stable carbon isotope measurements

Radiocarbon ages were measured on benthic and planktonic foraminifera from a series of cores recovered from the Brazil Margin by the R/V *Marion Dufresne* (MD09-3256Q, MD09-3257) and the Iberian Margin by the R/V *James Cook* (SHAK03-6K, SHAK05-3K, SHAK06-4K, SHAK10-10K and SHAK14-4G; Fig. 2). Results from these cores are combined with previously published data from the same transect regions (MD99-2334K and GS07-150-17/1GC-A)^22^,^34^. Core locations are given in Supplementary Table 1. Benthic stable carbon isotopes were also measured in these cores on *Cibicidoides wuellerstorfi*. No stable carbon isotopes were measured for SHAK10-10K because of the lack of any specimens of *Cibicidoides*.

Foraminifera were picked from the >212 μm size fraction and where necessary from the 150–212 μm fraction. Samples of *Globigerinoides ruber* (Brazil Margin) or *Globigerina bulloides* (Iberian Margin) and samples of mixed benthic foraminifera (excluding agglutinated species) were picked and graphitized in the Godwin Radiocarbon Laboratory at the University of Cambridge. Stable carbon isotope measurements were conducted on *Cibicidoides wuellerstorfi* and on DIC at the Godwin Laboratory, Cambridge.

### Radiocarbon measurements

Samples were cleaned on a glass plate with deionized water to remove any loose material. Samples were then dried and acidified. The CO_2_ produced was converted to graphite using a standard hydrogen/iron catalyst reduction method^35^. AMS-^14^C dates were obtained for the graphite samples at the ^14^Chrono Centre, Queens University Belfast. All dates are reported as conventional radiocarbon ages following^36^.

### Stable carbon isotope measurements on foraminifera

Stable carbon isotope measurements were conducted on *Cibicidoides wuellerstorfi* in the Godwin Laboratory. Each measurement was run on 1–3 individuals with a combined mass of 50–180 μg. The foraminifera were transferred into sample vials, crushed and then dried in an oven at 50 °C. Samples were reacted with orthophosphoric acid (100%) and the CO_2_ produced was cryogenically dried and then admitted to the dual inlet mass spectrometer for isotopic analysis by comparison with a reference gas. Each run of 30 samples was accompanied by 10 reference carbonates and 2 control samples. The results are reported with reference to the international standard VPDB and the precision is better than ±0.06‰ for ^12^C/^13^C and ±0.08‰ for ^16^O/^18^O.

### Stable carbon isotope DIC measurements

DIC measurements were made using a Thermo Gas Bench attached to a Delta V Mass Spectrometer. Three or four drops of orthophosphoric acid (100%) were preloaded into a reaction vial, which was capped, sealed and the headspace flushed with Helium gas. Approximately 1.5 ml of sample water was injected into the vial through the butyl rubber septa using a syringe and left to react for 1 h. The sample tubes were transferred to the Gas Bench and CTC CombiPal Autosampler and the resulting CO_2_ in the headspace analysed using a Thermo Delta V Mass Spectrometer. A series of standards and reference samples distributed throughout the run were used to calibrate to the international standard VPDB. Results have a reproducibility of better than 0.1 per mille.

### Ventilation ages

Here we report ventilation ages as either deep to shallow sub-surface radiocarbon age offsets, based on benthic-planktonic radiocarbon age differences (B-P), or as deep-atmospheric radiocarbon age offsets (B-Atm), which represent the B-P plus the surface reservoir age (that is, the shallow sub-surface versus atmosphere radiocarbon disequilibrium) that applies to the planktonic radiocarbon age. Although B-P offsets provide useful (and very accurate) information regarding vertical radiocarbon gradients at various locations in the ocean, B-Atm estimates are advantageous by virtue of referencing radiocarbon activities throughout the ocean interior relative to a single reference point: the radiocarbon activity of the atmosphere. They also maintain a constant scaling with respect to radiocarbon disequilibria, unlike relative isotopic offset metrics such as Δ^14^C (ref. 37). When mapped throughout the ocean, B-Atm offsets may thus provide a coherent means of inferring the patterns and rates of ocean-atmosphere CO_2_ exchange and transport in the ocean interior^38^. However, the uncertainty associated with LGM surface reservoir age estimates makes the derivation of accurate B-Atm offsets challenging.

### Surface reservoir ages at the LGM

To accurately reconstruct the distribution of radiocarbon in the Atlantic Ocean at the LGM, benthic radiocarbon activities must be referenced to a single (atmospheric) reference point. Therefore, we need to not only consider the deep- to surface age differences but also the surface to atmosphere age offsets that apply in each instance (alternatively each benthic radiocarbon date can be directly compared with the contemporary atmospheric radiocarbon age, given independent calendar ages). The planktonic foraminifera used in this study calcify in the upper surface ocean (<100 m deep), but even at these depths the water can be in significant disequilibrium relative to the atmosphere. Today surface reservoir ages range from around 400 to 600 years on the Brazil and Iberian Margins (GLODAP^38^). However, these would have varied in the past due to changes in *p*CO_2_ (affecting air–sea carbon isotope exchange at a given CO_2_ solubility), changes in ocean circulation (influencing the mixing/upwelling of ‘aged' waters into the surface ocean mixed layer) and, for example, changes in high latitude temperature, salinity and sea-ice cover (affecting CO_2_ solubility and gas exchange in sub- mixed-layer source regions). However, determining surface reservoir ages in the past is challenging, and not possible in contexts where independent calendar age or contemporaneous atmospheric radiocarbon age constraints are lacking. The modern surface reservoir age is therefore often used as a best guess, under the tentative assumption that physical conditions affecting radiocarbon exchange and transport have not changed over time^24^,^39^,^40^.

To accurately determine surface reservoir ages, a calendar age model that is independent of radiocarbon dating and a history of atmospheric radiocarbon variability are required. Although U/Th dating can be used to provide calendar ages for corals^43^, chronostratigraphic (including tephrochronological) approaches must be used for sediment cores. In high latitudes, cores can be linked stratigraphically to independently dated ice cores^21^,^22^,^41^. However, for low latitude, cores in which chronostratigraphic signals are typically more subdued and synchrony with high-latitude climate changes may be questionable, it is much more difficult to obtain an independent calendar chronostratigraphy. Yet, it is important to note that even at low latitudes, especially in regions with deep mixed layers or upwelling regimes, shallow sub-surface radiocarbon disequilibria are very likely to have changed across the last deglaciation^42^,^43^.

Although it is possible in principle to use model simulations to infer past surface reservoir ages, this approach requires knowledge of past changes in ocean circulation and ocean–atmosphere gas exchange efficiency (for example, because of changes in sea-ice extent or mixed layer depths), which strictly we do not have (indeed, this is typically what we are seeking). Despite this fundamental limitation, efforts have been made to model surface reservoir ages in the past, for example, assuming a constant ocean circulation^44^,^45^. These studies have shown that at the LGM the *p*CO_2_ difference alone would have caused surface reservoir ages to be around 250 years higher than under modern conditions—a passive response in the ocean that does not reflect the ocean's impact on atmospheric CO_2_ but rather the atmosphere's impact on the ocean. Additional changes in the mean exchange rate of CO_2_ between the ocean and the atmosphere, because of, for example, increased sea-ice cover and changes in the large-scale overturning circulation, are likely to have further increased these values, especially at high latitudes. Indeed, where shallow sub-surface reservoir age estimates are available for the LGM, they are typically significantly higher than the modern values^21^,^22^,^25^,^42^,^46^,^47^,^48^.

Despite the challenges involved in estimating past shallow sub-surface reservoir age variability, it is clear that ignoring them entirely is not a viable option, especially in a study that seeks to constrain past ocean circulation changes.

Recent work on core MD09-3257, from the Brazil Margin, showed that an independent calendar age model could be established for that core using U-Th dated speleothem records on the adjacent continent^49^, based on the fact that increased precipitation is marked by a decrease in δ^18^O in South American speleothems^50^,^51^ and increased sedimentary Ti/Ca ratios in marine sediment cores off the northeastern Brazilian coast^52^. However, because of a lack of available calendar age tie points for the LGM, surface reservoir ages based on this calendar age-scale remain sparse. We therefore adopt an estimate for LGM surface reservoir ages on the Brazil Margin based on the modern reservoir age corrected for *p*CO_2_-dependent air–sea gas exchange effects^44^,^45^. A reservoir age of 750 years (250 years greater than modern), which would have arisen due to globally reduced air–sea gas exchange rates, is used. Because it is unclear how ocean circulation changes may have affected this region we apply large uncertainties to this estimate of ±250 years. By combining high-resolution radiocarbon dating with calendar age models of centennial precision, it may be possible to reduce this uncertainty in the future.

Because the Iberian Margin cores contain a very clear event stratigraphy^53^,^54^, these cores were stratigraphically aligned to the uranium-series dated speleothem records from Hulu Cave^55^ and the layer counted (GICC05) NGRIP dust record^56^,^57^ using the Zr/Sr ratio determined using X-ray fluorescence (XRF; see Supplementary Figs 2 and 3). The cores were aligned to both the Hulu speleothem and NGRIP simultaneously using a series of tie points including two tie-points within the LGM (18–23 1000 years before present (kyrs BP)). Shallow sub-surface reservoir ages were determined directly by subtracting the planktonic age from the contemporaneous atmospheric age, using the calendar ages obtained at tie-points, and the atmospheric radiocarbon calibration curve IntCal13 (ref. 58; Supplementary Table 3). LGM surface reservoir ages on the Iberian Margin are thus estimated to be around 900 years with upper and lower limits of 1,100 years and 700 years, respectively. These ages are consistent with the lowest estimates determined for the Iberian Margin by ref. 22 (that is, excluding their tie-points in HS1 and the LGM where the event stratigraphy is arguably more equivocal).

### Age models

The planktonic radiocarbon ages were used to construct age models for each of the cores. In order to do so, an estimated surface reservoir age was subtracted from each planktonic radiocarbon date before conversion to calendar ages using BChron version 3.1.5 (ref. 59) and the IntCal13 calibration curve^58^. These calendar ages were then used to construct sediment depth-age models using the Markov Chain Monte-Carlo method, also using Bchron. As LGM surface reservoir age estimates have not been directly estimated on the Brazil Margin, a ‘best guess' value of 750 years, based on isotope exchange constraints backed up by model simulations^44^,^45^, was used. For the Iberian Margin, a surface reservoir age of 900 years was used for the LGM based on the stratigraphic alignments described above.

For compiled data, for cores with age models that do not depend on radiocarbon data, surface and deep reservoir ages were determined by subtracting the contemporary atmospheric radiocarbon age (based on Intcal13) from the planktonic and benthic radiocarbon ages, respectively. For sites with no independent age model, the *p*CO_2_ corrected modern reservoir ages (that is, modern plus 250 years at the LGM) were used and added to the benthic-planktonic age offset to determine the deep ocean-atmospheric age offset (Supplementary Table 2).

### Stratigraphic alignments

The cores were stratigraphically aligned to the Hulu speleothem δ^18^O record^55^ and the NGRIP dust record^57^ (on the GICC05 age model^56^,^60^). The NGRIP dust content changes rapidly and synchronously with changes in ice core δ^18^O (ref. 61). NGRIP dust, which is predominantly sourced from East Asian deserts^62^,^63^,^64^,^65^, increases during cold periods^57^. These increases are caused by atmospheric changes^66^,^67^ and/or intensified sources^68^, but the exact mechanism is unconstrained. The Hulu speleothem δ ^18^O record resembles East Asian monsoon changes and is highly correlated with millennial scale Greenland temperature variations^55^,^69^.

The sediment cores were aligned using elemental ratios determined using high-resolution XRF. Zr/Sr ratios were used to align the Iberian Margin cores. Zr/Sr anti-correlates strongly with Ca/Ti in these cores. The Zr/Sr and Ca/Ti ratios reflect the relative input of biogenic (Ca, Sr) and detrital (Zr, Ti) material, and have been shown to correlate strongly with planktonic δ ^18^O and alkenone sea-surface temperatures on the Iberian Margin, as well as with millennial scale variations in Greenland ice core δ ^18^O (ref. 70). Zr/Sr values are generally higher during stadials and lower during interstadials, whereas Ca/Ti ratios show the opposite trend.

A master core, SHAK03-6K was selected for the Iberian Margin through which the other cores were aligned. SHAK03-6K was chosen as the master core based on its high quality (no discontinuities) and age range (SHAK14-4G was also of very high quality but only reached back to ∼20 kyrs). SHAK03-6K was aligned to both the Hulu speleothem and the NGRIP dust record using a series of tie points (Supplementary Fig. 2). No tie-points were used in the interval 0–11.4 kyrs BP because of the lack of clear signals in the records. The calendar ages at tie points in SHAK03-6K were then transferred to all the other Iberian Margin cores (Supplementary Fig. 3).

### XRF data

The Iberian Margin cores were scanned at the University of Cambridge using an Avaatech XRF core scanner (2nd generation). The surface of the cores was scraped clean then covered with a 4-μm SPEXCertiPrep Ultralene foil to avoid contamination and to prevent the cores drying out and cracking. Each section was measured at three different voltages and currents: 10 kV and 750 μA, 30 kV and 500 μA, and at 50 kV and 1,000 μA. The entire length of each core was analysed at 5-mm resolution with an irradiated surface length and width of 5 mm (downcore) and 12 mm (cross core), respectively. The count time was 60 s for each measurement. Element intensities were obtained by post-processing of the XRF spectra using the Canberra WinAxil software with standard software settings and spectrum-fit models.

### Data availability

The data supporting the findings of this study are available within the article and its Supplementary Information files.

## Additional information

**How to cite this article:** Freeman, E. *et al.* Radiocarbon evidence for enhanced respired carbon storage in the Atlantic at the Last Glacial Maximum. *Nat. Commun.* 7:11998 doi: 10.1038/ncomms11998 (2016).

## Supplementary Material

Supplementary Information Supplementary Figures 1-3, Supplementary Tables 1-4 and Supplementary References.
