# Supplementary material for: Radiocarbon evidence for enhanced respired carbon storage in the Atlantic at the Last Glacial Maximum
Source: Nat Commun. 2016 Jun 27;7:11998. doi: 10.1038/ncomms11998 (PMC4931225; doi:10.1038/ncomms11998)
Supplement: Supplementary Information — Supplementary Figures 1-3, Supplementary Tables 1-4 and Supplementary References. [file ncomms11998-s1.pdf]

Supplementary Figure 1:

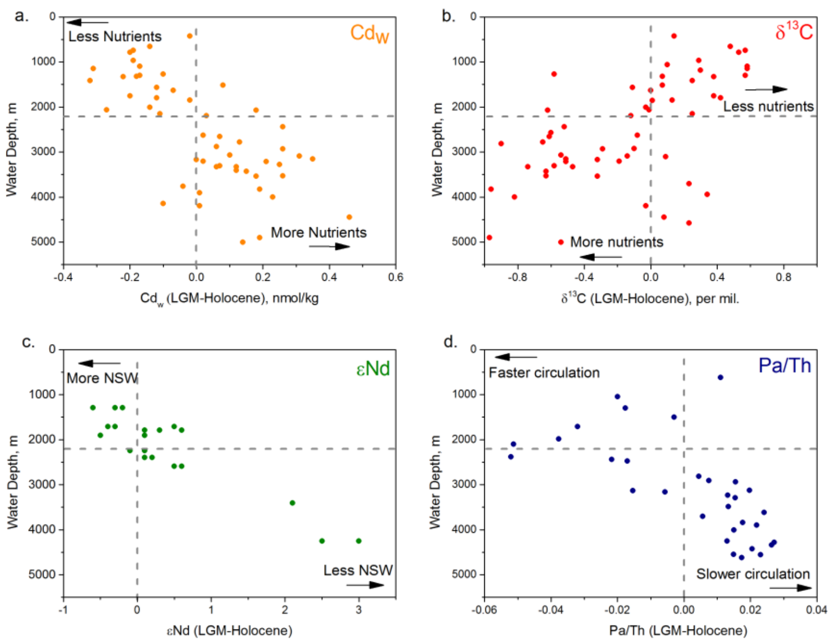

LGM-Holocene depth profiles in the Atlantic Ocean showing an opposite sense of change above and below around 2.2km in various proxies: a) Cd_W of benthic foraminifera[1], b) $\delta^{13}C$ measured on *Cibicidoides*[1], c) Authigenic Nd isotope signal[2], d) $^{231}Pa/^{230}Th$ data[3].

Supplementary Figure 2:

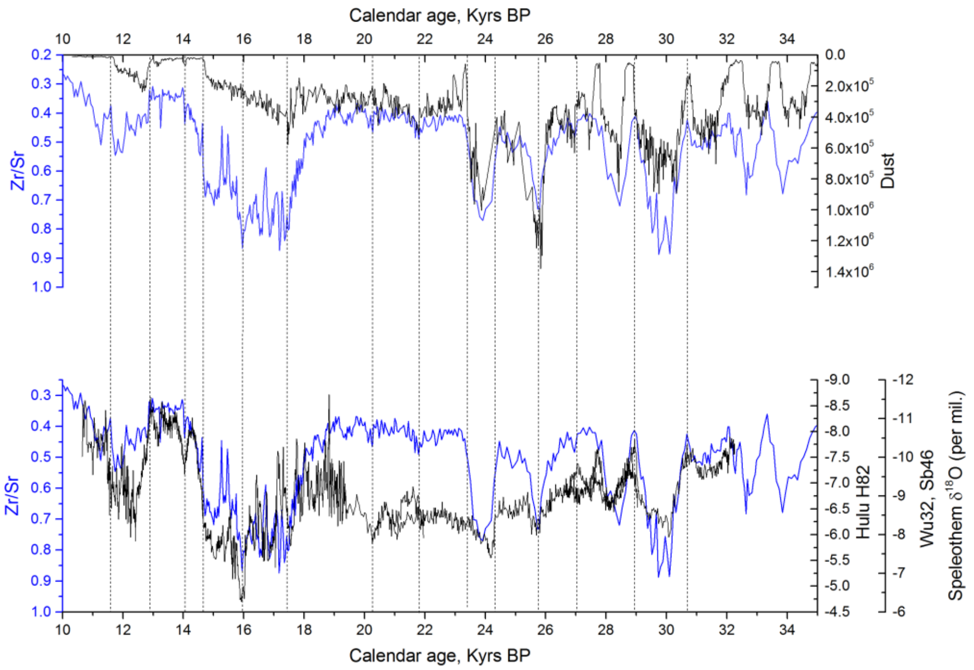

Stratigraphic alignment of SHAK03-6K to the NGRIP dust record[4] (above) on GICCO5 age model[5,6] and the Hulu speleothem $\delta^{18}O$ record[7] (below). Tie points are indicated by the vertical dashed lines.

Supplementary Figure 3:

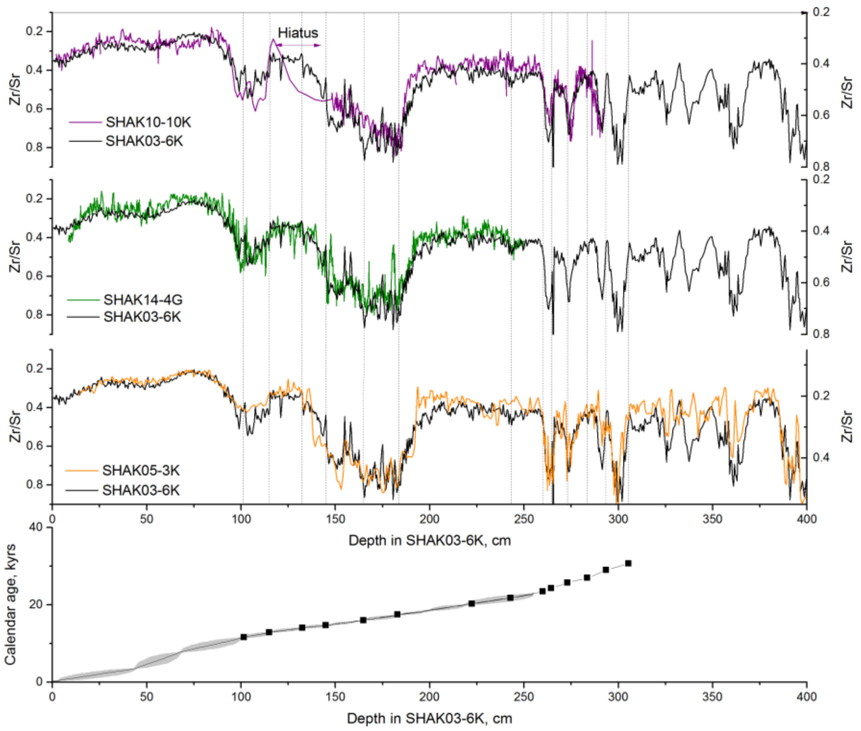

Upper panel: Stratigraphic alignment of top) SHAK10-10K; middle) SHAK14-4G and bottom) SHAK05-3K on to the master core, SHAK03-6K. Lower panel: Age-depth profile for SHAK03-6K. Black squares and line indicate depths and calendar ages at the stratigraphic tie points. Grey line represents the calendar ages obtained using Bchron (95% confidence shaded grey).

Supplementary Table 1: Locations of cores in this study

Core name	Location	Latitude	Longitude	Water Depth
MD09 3257	Brazil Margin	04°14.68’ S	36°21.16’ W	2344
MD09 3256Q	Brazil Margin	03°32.81’ S	35°23.11’ W	3537
JC89-SHAK-10-10K	Iberian Margin	37°50.00’ N	09°30.65’W	1127
JC89-SHAK-14-4G	Iberian Margin	37°50.16’ N	09°43.61’W	2063
JC89-SHAK-06-4K	Iberian Margin	37°33.68’ N	10°21.89’W	2642
JC89-SHAK-03-6K	Iberian Margin	37°42.54’ N	10°29.56’W	3735
JC89-SHAK-05-3K	Iberian Margin	37°36.26’ N	10°41.50’W	4670

Supplementary Table 2: Surface reservoir ages on the Iberian Margin. Ages are determined at tie-points only due to uncertainties in calendar age between the tie points.

Core	Tie depth, cm	Tie age, Calendar yrs BP	Interpolated planktic 14C age, 14C yrs	Atmospheric 14C age, 14C yrs	Reservoir age, 14C yrs
SHAK03-6K	222.5	20262	17404	16802	602
SHAK05-3K	140.2	20262	17787	16802	984
SHAK10-10K	242.3	20262	17810	16802	1007
SHAK14-4G	453.4	20262	17514	16802	711
MD99-2334K	291.3	20262	17726	16802	924
SHAK03-6K	243	21783	18782	17947	835
SHAK05-3K	152.4	21783	18864	17947	917
SHAK10-10K	277.6	21783	-	-	-
SHAK14-4G	497.8	21783	18817	17947	870
MD99-2334K	314.0	21783	19167	17947	1220
				**Average**	**897**
				**Standard deviation**	**177**

Supplementary Table 3: Radiocarbon ages of benthic and planktonic foraminifera samples from the LGM. Benthic samples are mixed species (excluding agglutinated) and planktonic samples are *G.ruber* (Brazil Margin) or *G.bulloides* (Iberian Margin).

Core	Depth in core	Planktic 14C age	Error	Benthic 14C age	Error	B-P	Error
SHAK10-10K	252	18166	90	17707	74	-459	117
SHAK14-4G	432	16886	75	17488	82	602	111
SHAK14-4G	488	18531	89	18975	87	444	124
SHAK06-4K	210	17508	97	18409	120	901	154
SHAK06-4K	250	18950	106	20195	145	1245	180
SHAK03-6K	219	17169	86	18378	95	1209	128
SHAK03-6K	255	19589	144	21034	145	1445	204
SHAK05-3K	132	17144	91	18661	141	1517	168
SHAK05-3K	160	19536	136	21031	155	1495	206
MD09-3257	189	16860	141	16252	87	608	166
MD09-3257	193	17829	97	16675	90	1154	132
MD09-3256	62	17303	75	16159	65	1144	99
MD09-3256	66	18355	118	17385	105	970	158
MD09-3256	68	20258	164	18544	159	1714	228
GS07-150-17/1	162	17110	105	16783	86	327	136
GS07-150-17/1	180	19783	118	19658	136	125	180

Supplementary Table 4: Compiled radiocarbon ventilation ages at the LGM in the Atlantic Ocean[8–15]

Location	Depth	B-P	Error	Res. Age	Error	B-atm	Reference
Brazil Margin	1000	226	113	750	250	976	This study
Brazil Margin	2344	881	106	750	250	1631	This study
Brazil Margin	3537	1276	98	750	250	2026	This study
Western North Atlantic	2975	1145	85	750	250	1895	Keigwin and Schlegel, 2002 (8)
Western North Atlantic	3845	1000	170	750	250	1750	Keigwin et al., 2004 (9)
Western North Atlantic	4250	1550	120	750	250	2300	Keigwin et al., 2004 (9)
Western North Atlantic	4712	1450	170	750	250	2200	Keigwin et al., 2004 (9)
Iberian Margin	1127	-459	117	900	200	441	This study
Iberian Margin	2063	523	83	900	200	1423	This study
Iberian Margin	2642	1073	118	900	200	1973	This study
Iberian Margin	3735	1327	120	900	200	2227	This study
Iberian Margin	4670	1506	133	900	200	2406	This study
Iberian Margin	3146	1510	189	900	200	2410	Skinner et al., 2014 (10)
Eastern Equatorial Atlantic	550	240	85	585	300	825	Cleroux et al., 2011 (11)
South Atlantic	1268	648	48	750	250	1398	Sortor and Lund, 2011 (12)
South Atlantic	3770	1635	94	1842	300	3477	Skinner et al., 2010 (13)
South Atlantic	4981	1063	69	1320	300	2383	Barker et al., 2010 (14)
Drake Passage	819	-	-	-	-	1697	Burke and Robinson, 2012 (15)
Drake Passage	1134	-	-	-	-	1680	Burke and Robinson, 2012 (15)

Supplementary references:

1. Marchitto, T. M. & Broecker, W. S. Deep water mass geometry in the glacial Atlantic Ocean: A review of constraints from the paleonutrient proxy Cd/Ca. *Geochem. Geophys. Geosystems* **7,** Q12003 (2006).
2. Gutjahr, M., Frank, M., Stirling, C. H., Keigwin, L. D. & Halliday, A. N. Tracing the Nd isotope evolution of North Atlantic Deep and Intermediate Waters in the western North Atlantic since the Last Glacial Maximum from Blake Ridge sediments. *Earth Planet. Sci. Lett.* **266,** 61–77 (2008).
3. Lippold, J. *et al.* Strength and geometry of the glacial Atlantic Meridional Overturning Circulation. *Nat. Geosci.* **5,** 813–816 (2012).
4. Ruth, U., Wagenbach, D., Steffensen, J. P. & Bigler, M. Continuous record of microparticle concentration and size distribution in the central Greenland NGRIP ice core during the last glacial period. *J. Geophys. Res. - Atmospheres* **108,** 4098 (2003).
5. Rasmussen, S. O. *et al.* A new Greenland ice core chronology for the last glacial termination. *J. Geophys. Res. - Atmospheres* **111,** D06102 (2006).
6. Andersen, K. K. *et al.* The Greenland Ice Core Chronology 2005, 15ka. Part 1: constructing the time scale. *Quat. Sci. Rev.* **25,** 3246–3257 (2006).
7. Southon, J., Noronha, A. L., Cheng, H., Edwards, R. L. & Wang, Y. A high-resolution record of atmospheric 14C based on Hulu Cave speleothem H82. *Quat. Sci. Rev.* **33,** 32 (2012).
8. Keigwin, L. D. & Schlegel, M. A. Ocean ventilation and sedimentation since the glacial maximum at 3 km in the western North Atlantic. *Geochem. Geophys. Geosystems* **3,** 1034 (2002).

9. Keigwin, L. D. Radiocarbon and stable isotope constraints on Last Glacial Maximum and Younger Dryas ventilation in the western North Atlantic. *Paleoceanography* **19,** PA4012 (2004).
10. Skinner, L. C., Waelbroeck, C., Scrivner, A. E. & Fallon, S. J. Radiocarbon evidence for alternating northern and southern sources of ventilation of the deep Atlantic carbon pool during the last deglaciation. *Proc. Natl. Acad. Sci. U. S. A.* **111,** 5480 (2014).
11. Cléroux, C., Demenocal, P. & Guilderson, T. Deglacial radiocarbon history of tropical Atlantic thermocline waters: absence of CO_2 reservoir purging signal. *Quat. Sci. Rev.* **30,** 1875–1882 (2011).
12. Sortor, R. N. & Lund, D. C. No evidence for a deglacial intermediate water [DELTA]^{14}C anomaly in the SW Atlantic. *Earth Planet. Sci. Lett.* **310,** 65 (2011).
13. Skinner, L. C., Fallon, S., Waelbroeck, C., Michel, E. & Barker, S. Ventilation of the Deep Southern Ocean and Deglacial CO_2 Rise. *Science* **328,** 1147–1151 (2010).
14. Barker, S., Knorr, G., Vautravers, M. J., Diz, P. & Skinner, L. C. Extreme deepening of the Atlantic overturning circulation during deglaciation. *Nat. Geosci.* **3,** 567–571 (2010).
15. Burke, A. & Robinson, L. F. The Southern Ocean's role in carbon exchange during the last deglaciation. *Science* **335,** 557–561 (2012).
